# Inhibitor of PD-1/PD-L1: a new approach may be beneficial for the treatment of idiopathic pulmonary fibrosis

**DOI:** 10.1186/s12967-024-04884-7

**Published:** 2024-01-23

**Authors:** Jie Tan, Qianfei Xue, Xiao Hu, Junling Yang

**Affiliations:** 1 Department of Respiratory Medicine, The Second Hospital of Jilin University, Changchun, China; 2 Hospital of Jilin University, Changchun, China

**Keywords:** PD-1, PD-L1, Immune checkpoint, Idiopathic pulmonary fibrosis, Mechanism of action, Treatment, Lung cancer

## Abstract

Idiopathic pulmonary fibrosis (IPF) is a globally prevalent, progressive disease with limited treatment options and poor prognosis. Because of its irreversible disease progression, IPF affects the quality and length of life of patients and imposes a significant burden on their families and social healthcare services. The use of the antifibrotic drugs pirfenidone and nintedanib can slow the progression of the disease to some extent, but it does not have a reverse effect on the prognosis. The option of lung transplantion is also limited owing to contraindications to transplantation, possible complications after transplantation, and the risk of death. Therefore, the discovery of new, effective treatment methods is an urgent need. Over recent years, various studies have been undertaken to investigate the relationship between interstitial pneumonia and lung cancer, suggesting that some immune checkpoints in IPF are similar to those in tumors. Immune checkpoints are a class of immunosuppressive molecules that are essential for maintaining autoimmune tolerance and regulating the duration and magnitude of immune responses in peripheral tissues. They can prevent normal tissues from being damaged and destroyed by the immune response. While current studies have focused on PD-1/PD-L1 and CTLA-4, PD-1/PD-L1 may be the only effective immune checkpoint IPF treatment. This review discusses the application of PD-1/PD-L1 checkpoint in IPF, with the aim of finding a new direction for IPF treatment.

## Introduction

Idiopathic pulmonary fibrosis (IPF) is a specific manifestation of progressive chronic pulmonary interstitial pneumonia of unknown origin and mainly occurs in the elderly [1]. Studies have shown that the incidence of IPF is increasing globally [2], and increases with age [3]. The primary clinical manifestations involve progressive and deteriorating dyspnea [4], and the diagnosis of IPF typically relies on histology and high-resolution computed tomography of the chest [5]. The characteristic pathological features of IPF include characteristic fibroblast foci, marked fibrosis, or honeycomb-shaped lungs [6]. Previous research suggests that the pathogenesis of IPF is associated with abnormally activated alveolar epithelial cells (AECs), which produce a variety of cytokines and growth factors, stimulate epithelial cell transformation into mesenchymal cells, and induce the formation of fibroblasts and myofibroblast foci. These fibroblasts and myofibroblast foci secrete excessive extracellular matrix (ECM), leading to scarring and lung remodeling [7]. The molecular mechanism of IPF is still poorly understood. However, studies have shown that transforming growth factor (TGF)-β signaling plays a central role in fibrosis progression [8]. However, since IPF has no specific clinical manifestations and most patients do not undergo relevant examinations in the early stage of the disease, the diagnosis and treatment of IPF pose a great challenge. Further, the course of the disease is irreversible, resulting in severe physical, psychological, and socio-economic burdens [9].

The current first-line protocol for diagnosing progressive IPF is the use of the antifibrotic drugs pirfenidone and nintedanib [10, 11]. Although the use of antifibrotic drugs can slow the deterioration of forced vital capacity in some patients, it does not extend survival time after diagnosis [12] and has a relatively limited effect on improving lung function and quality of life in patients with end-stage IPF [10, 11]. Lung transplantation, currently the only treatment for IPF that can improve symptoms and prolong survival, may only be used in a small number of patients with IPF [13]. Whether patients with IPF can undergo lung transplantation must be evaluated considering age, illness, comorbidities, contraindications, risk of death, and complications after transplantation [14]. Therefore, the need is urgent for treatments that can extend the lives of patients with IPF and improve their quality of life. Unfortunately, current treatments do not meet these requirements.

IPF has also been reported to share common risk factors with cancer [15]. Some articles have summarized the mechanisms underlying the interaction between IPF and lung cancer (LC), indicating that there are indeed some epidemiological, mechanistic, and genetic associations [16, 17]. One study summarized the reported incidence of LC in patients with IPF in recent years, which ranged from 2.7 to 48% [17], much higher than the incidence of LC in the general population [18]. Considering the association between these two diseases, studies have begun to test known anticancer drugs against IPF [19], with a focus on immune checkpoints.

PD-1/PD-L1 is involved in immune regulation and in maintaining the immune tolerance to autoantigens via a mechanism that is likely to be associated with the inhibition of T-cell hyperactivation and cytokine secretion [20]. Inhibiting the PD-1/PD-L1 axis can reverse the immunosuppressive targeting of the tumor microenvironment and restore the antitumor effects of T cells. Previous studies have found that PD-L1 is highly expressed in invasive lung fibroblasts and drives the progression of lung fibrosis in humanized mice [21]. Knocking down PD-L1 expression can also reduce TGF-β-induced extracellular matrix production in human and mouse lung fibroblast cell lines [22]. Other studies have suggested that PD-L1 can promote pulmonary fibrosis through the Smad3/β‑catenin pathway [23]. The role of the PD-1/PD-L1 immune checkpoint in IPF and the possible therapeutic effect of inhibiting this immune checkpoint on IPF still need to be further explored. In this review, we explored the potential of PD-1/PD-L1 inhibitors in the treatment of IPF based on current studies on PD-1/PD-L1 immune checkpoint.

### Co-pathogenic mechanism and signaling pathways of IPF and LC

Potential risk factors for IPF include genetic mutations, viral infections, lifestyle choices, environmental exposures, and occupational hazards [24]. Smoking, air pollution, and occupational exposure are common risk factors for IPF and LC [25, 26]. The shared pathogenesis of IPF and LC [17, 27] includes uncontrolled cell proliferation [28] and interference with intercellular transmission [29, 30]. Studies have shown that certain cells (e.g. myofibroblasts, cancer-associated fibroblasts [CAFs]) and associated growth factors are equally involved in the disease development of IPF and LC [31]. Of these, myofibroblasts are particularly important in IPF because of their proliferative capacity and their ability to be activated under high fibrotic conditions. These abilities enable myofibroblasts to secrete and deposit ECM proteins, leading to lung fibrosis [32]. TGF-β1 induces the conversion of epithelial cells from epithelial-to-mesenchymal transition (EMT) to fibroblasts/myofibroblasts, thereby promoting fibrous proliferation. Furthermore, alveolar type II epithelial cells have been observed to differentiate into fibroblasts/myofibroblasts and produce the ECM under TGF-β1 stimulation [33].

CAFs are key cellular actors in LC, especially in non-small cell LC (NSCLS) [34]. Several hypotheses have been proposed regarding the cellular origin of CAF in tumors [16], as follows: resident fibroblasts differentiate into CAFs during tumor development under the coordination by certain signaling pathways and cancer-associated adipocytes, which exist in tumor stroma, possibly originating from circulating progenitor cells [35]; bone marrow MSCs and hematopoietic stem cells [36]; epithelial cells via EMT [37]; and vascular endothelial cells are critical to tumor angiogenesis via endothelial-to-mesenchymal transformation [38]. Therefore, TGF-β not only regulates the major molecules that promote fibrosis signaling but also promotes LC progression and cancer cell mitosis. In addition to the mesenchymal phenotype, cell transformation [27, 39] represents a common association between pulmonary fibrosis and carcinogenesis.

**Fig. 1:**
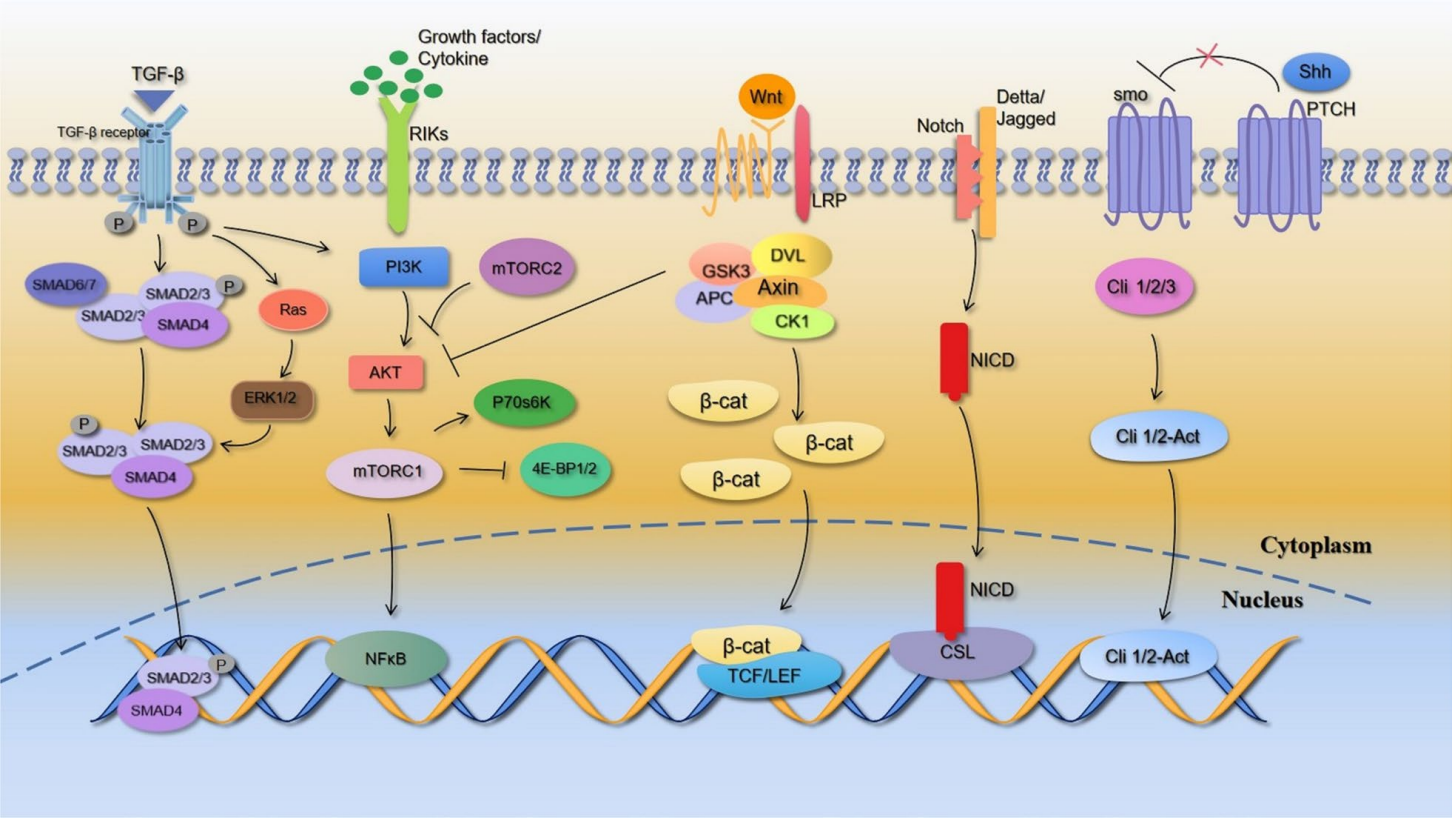
Five common pathways in lung cancer and IPF. These common signaling pathways in IPF and lung cancer are involved in the regulation of cell proliferation, apoptosis, differentiation, and migration. The TGF-β/Smad pathway is involved in ECM deposition and EMT processes in tissue fibrosis, whereas the PI3K/AKT/mTOR pathway is associated with fibroblast differentiation and cancer metastasis and migration. The Wnt/β-catenin pathway is associated with tissue invasion, while the Notch pathway mediates EMT in fibroblasts, promoting proliferation and inhibiting apoptosis in cancer cells. The Shh pathway promotes epithelial cell susceptibility to apoptosis and enhances fibroblast resistance to apoptosis

Abnormal activation of the following common signaling pathways (Fig. 1) has been reported to occur in both pulmonary fibrosis and lung cancer:

#### TGF-β/Smad pathway

This key pathway for fibrogenesis involves TGF-β1 being responsible for the activation of fibroblasts into myofibroblasts [40] and being classically signaled by the SMAD protein, which is a key pathway for fibrogenesis [41, 42]. TGF-β promotes ECM deposition, or selectively inhibits collagenase synthesis, induces the production of collagenase inhibitors or inhibits the expression and activation of protease inhibitors, and ultimately reduces collagen degradation, thus effectively promoting the formation of ECM-mediated pulmonary fibrosis [43]. In early-phase LC, TGF-β induces the arrest and apoptosis of normal and malignant cancer cells, thereby inhibiting tumor growth. Conversely, TGF-β also stimulates tumor EMT to promote tumorigenesis when cancer cells lose their oncogenic function or undergo mutations [44].

#### Wnt/β-catenin pathway

This pathway induces EMT by regulating certain cytokines associated with tissue infiltration. In lung fibrosis, the classical pathway of tracheal injury-induced alveolar type II epithelial cells is the activation of this signaling pathway to promote the activation of mesenchymal fibroblasts; sustained stimulation eventually leads to pulmonary fibrosis. Activation of this pathway also promotes the conversion of perivascular fibroblasts into myofibroblasts, leading to ECM accumulation and pulmonary fibrosis development [45]. The expression of the pathway-related protein TCF/LEF considerably increases following the activation of the Wnt signaling pathway in LC, thereby activating the transcription of Sox-2, c-Myc, cyclin D1, and other downstream target genes [46].

#### PI3K/AKT/mTOR pathway

Dysregulation of this pathway is involved in cell proliferation and apoptosis. TGF-β can activate the PI3K/Akt pathway by activating extracellular signals in addition to the Smad pathway [47]. In pulmonary fibrosis, PI3K was observed to be associated with fibroblast proliferation and differentiation [48]. Similarly, dysregulation of this signaling pathway has been observed in NSCLC and is primarily associated with cancer metastasis and invasion [49].

#### Notch pathway

Reactivation of the Notch pathway is essential for EMT in fibroblasts and it also promotes α-smooth muscle actin expression in fibroblasts [50]. This pathway is also involved in the NSCLC process, and in addition to stimulating NSCLC cell propagation and inhibiting NSCLC cell apoptosis, members of the Notch signaling pathway may also be able to predict disease progression and prognosis in patients with NSCLC [51]. Furthermore, activation of the Notch pathway in subpopulations of tumor cells in small cell lung cancer (SCLC) contributes to the establishment of the tumor microenvironment [52].

#### Sonic hedgehog (Shh) pathway

Over-expression of the Shh pathway promotes apoptosis in epithelial cells while enhancing fibroblast resistance to apoptosis [53]. In the development of early lung cancer, tumor stem cells can activate the Shh pathway and affect other tumor cells through paracrine effects, leading to tumor proliferation, spread, and EMT [54].

Gene set enrichment analysis of the IPF and NSCLC datasets revealed a common pattern of gene dysregulation [55]. However, IPF and NSCLC differ considerably at the global transcriptome level, with only a limited number of key genes exhibiting similar regulation, so successful treatment options for patients with cancer may provide unexpected benefits for patients with IPF [56]. The emergence of immune checkpoint inhibitors (ICIs) has provided a reliable and bright prospect for the immunotherapy of cancer.

### Expression of PD-1/PD-L1 in patients with IPF

PD-1 is an immunosuppressive molecule mainly expressed in activated T cells and can be enhanced through stimulation with tumor necrosis factor [57]. PD-L1 is the main ligand of PD-1 and is widely expressed in T cells, B cells, dendritic cells, macrophages, and other tissues [58]. Blocking negative regulatory signaling then reactivates T-cell activity and promotes tumor cell killing ability, which is mainly achieved by blocking PD-1 binding to PD-L1 [59]. ICIs primarily exert their antitumor effects by blocking tumor cell escape, helping in repositioning the immune cells and engaging in cytotoxic killing [60]. In particular, PD-1/PD-L1 inhibitors block PD-1 on T cells or PD-L1 on the surface of tumor cells, leading to antitumor effects [61]. At present, PD-1/PD-L1 inhibitors have been approved for the treatment of patients with advanced NSCLC, hepatocellular carcinoma, urothelial carcinoma, metastatic melanoma, and metastatic colorectal cancer [62]. The clinical benefit of PD-1/PD-L1 inhibitors is usually predicted by immunohistochemical determination of PD-L1 expression levels [63]; high expression (> 1 or > 50% in NSCLC) is associated with better response rates [64, 65]. Given the similar pathogeneses and pathway mechanisms of IPF and cancer, we wondered whether ICIs could benefit patients with IPF.

The methods frequently employed to detect PD-1/PD-L1 expression in human specimens include enzyme-linked immunosorbent assay (ELISA) [66], flow cytometry [67], and immunofluorescence [23]. The results of these studies are summarised in Table 1.

**Table 1:** Summary of clinical studies of PD-1/PD-L1 in human specimens

Reference setting	PD-1/PD-L1	IPF patients/normal or other disease numbers	Sample	Method	Expression change
Jovanovic [66]	mPD-L1 sPD-L1	12/– 23/–	Lung Plasma	IHC LISA	Upregulation Elevation
Celada [67]	PD-1 PD-L1	10/5 25/24 10/5	Lung Peripheral blood	IHC FCM IHC	Upregulation Upregulation Upregulation
Guo [23]	PD-L1	3/3	Lung	IF	Upregulation
Cui [76]	PD-L1	11/3	Lung	Mass cytometry	Upregulation
Bonham [68]	PD-1	41/13	Peripheral blood	FCM	No change
Wang [69]	PD-1 PD-L1	23//25 23//25	Peripheral blood Peripheral blood	FCM	Upregulation Upregulation
Milenkovic [70]	sPD-L1	30/30	Plasma	ELISA	Elevation
Guyard [71]	PD-L1	18/13 (all complicated lung cancer)	Lung	IHC	Upregulation
Kronborg-White [72]	mPD-L1	43/55	Lung	IHC	Upregulation
Asai [73]	PD-1	18/21	Peripheral blood	FCM	Upregulation
Li [74]	PD-L1	16/8 8/10 12/12	Lung BALF Serum	IHC ELISA ELISA	Upregulation Elevation Elevation
Ahmadvand [75]	CD274 PD-1	9/12	Lung	FCM/RT-PCR	Upregulation Upregulation

*IPF* idiopathic pulmonary fibrosis, *PD-1* programmed cell death 1, *PD-L1* programmed death-ligand 1, *IF* immunofluorescence, *FCM* flow cytometry, *mPD-L1* membrane programmed death-ligand 1, *sPD-L1* soluble programmed death-ligand 1, *IHC* immunohistochemistry, *WB* Western blot, *ELISA* enzyme-linked immunosorbent assay, *Th17* T helper 17, *Tfh cells* T follicular helper cells, *AT2* Alveolar epithelial type 2 cells

One study reported significantly elevated levels of PD-L1 expression in the serum of IPF patients [66], while another study showed the opposite, with no change in PD-L1 expression in the peripheral blood of IPF patients [68]. However, although no alterations were observed in PD-L1 expression in peripheral blood, PD-1 levels in circulating CD4 + T cells and lung tissue in IPF patients were observed to be higher than in age-matched healthy controls in another previous study [67]. Another study observed increased expression of PD-1 and PD-L1 in CD4 + T cells in the peripheral blood of patients [69]. In addition to the determination of PD-L1 expression, studies have also observed changes in the expression of soluble PD-L1 (sPD-L1), with one study showing significantly higher sPD-L1 levels in the serum of IPF patients without surgical biopsy than in healthy controls [66], and another study observing a similar presentation [70]. Given the similarity between IPF and NSCLC, a previous study used lung tissue from patients with lung fibrosis-related cancers as a sample to explore changes in PD-L1 expression [71]. Another previous study showed that PD-L1 was highly expressed on the cell membranes of alveolar and bronchial epithelial cells in lung tissue samples from patients with IPF and pulmonary fibrosis [72]. Alternatively, other studies reported that the PD-L1 expression was not significantly altered in fibroblasts and myofibroblasts in the lung tissues of patients but was overexpressed in alveolar macrophages [66]. Asai et al. reported a significantly higher proportion of PD-1^+^ICOS^+^Tfh cells in patients with IPF [73]. Li et al. observed elevated levels of PD-L1 in lung tissue, alveolar lavage fluid, and serum from patients [74]. Ahmadvand et al. came to the same conclusion for lung tissue and reported that PD-1 was highly expressed in T cells [75].

We noticed that in this table, Bonham et al. [68]found that the expression level of PD-1 did not increase as it did in other studies, which may be related to the fact that the samples used were fresh peripheral blood PBMC, the control group was age-sex matched, and the average fluorescence intensity of PD-1 expression was used in data analysis. Celada et al. [67] and Wang et al. [69] reported the percentage of PD-1^+^CD4^+^ T cells, while Asai et al. [73] reported the proportion of PD-1^+^ICOS^+^Tfh cells in peripheral blood. These results suggest that PD-1/PD-L1 is involved in the process of pulmonary fibrosis. Nonetheless, all studies published to date have been retrospective in design and have small sample sizes; therefore, further research is required. Prospective cohort studies can be designed for further exploration, with as large a sample size as possible, and care should be taken to eliminate bias and confounding factors in the study. Prospective cohort studies can be designed to explore further, with as large a sample size as possible, in addition to being aware of bias and confounding factors in the studies.

### Preclinical studies of PD-1/PD-L1 in pulmonary fibrosis

The potential role of the PD-1/PD-L1 immune checkpoint in IPF was observed, and a series of preclinical studies were initiated in the hope that this new anti-tumor drug could bring a promising future to the treatment of IPF. The role of PD-1/PD-L1 in the pathogenesis of IPF has been explored through in vivo and in vitro experiments. Table 2 summarizes the results of these studies in animal models of IPF.

**Table 2:** Summary of clinical studies of PD-1/PD-L1 in mice specimens

Reference setting	PD-1/PD-L1	Exposure	Sample	Cell type	Method	Expression change
Celada [67]	PD-1	Bleomycin	Lung	CD4^+^ T cells	FCM	Upregulation
Guo [23]	PD-L1	Bleomycin	Lung	Fibroblasts	IF	Upregulation
Cui [76]	PD-L1	Bleomycin	Lung	Fibroblasts	Mass cytometry IF/FCM	Upregulation
		Primary human fibrotic lung fibroblast engraftation	Lung	Fibroblasts		Upregulation
	PD-1	Bleomycin	Lung	CD8^+^ T cells	Mass cytometry	Upregulation
Li [74]	PD-L1	Bleomycin paraquat	Lung Lung		IHC/WB IHC/WB	Upregulation Upregulation
Geng [21]	PD-L1	CD274^high^ and CD274^low^ IPF lung normal fibroblasts injection	Lung	Fibroblasts	PCR RNA-seq	Upregulation
Lu [77]	PD-L1	Bleomycin	Lung	Fibroblasts	WB/IHC	Upregulation
Wang [78]	PD-1	Bleomycin	Lung	/	IHC	Upregulation
			Peripheral blood	CD4^+^ T cells	FCM	Upregulation
Zhao [83]	PD-1	Silica	Lung	/	WB/IHC FCM/PCR	Upregulation
	PD-L1		Lung	/		Upregulation

*PD-1* programmed cell death 1, *PD-L1* programmed death-ligand 1, *IF* immunofluorescence, *FCM* flow cytometry, *IHC* immunohistochemistry, *WB* Western blot, *PCR* polymerase chain reaction, *RNA-seq* RNA sequencing

In three studies on mouse models of lung fibrosis, elevated levels of PD-L1 expression in lung tissue lesions were observed [23, 76, 77]. Furthermore, aberrant PD-1 expression was observed in a mouse model of pulmonary fibrosis [67, 76, 78]. Celada et al. observed elevated levels of PD-1 in CD4^+^ T cells from the lung tissue of fibrotic mice [67], whereas Cui et al. observed this phenomenon in CD8^+^ T cells [76]. In addition to the elevated expression of PD-1 observed in the lung tissue of fibrotic mice, Wang et al. found elevated expression of this indicator in CD4 + T cells of the peripheral blood of mice [78]. However, none of the above studies measured the specific expression levels of PD-1.

Based on research on human tissue transplantation, increasingly more immunodeficient mice have been used as preclinical animal models over recent years [66, 79–81]. Some studies have also used humanized mouse models for PD-1/PD-L1 immune checkpoint related studies [21, 76, 82]. Geng et al. used NOD^−^SCID^−^IL2Rgc^−/−^ (NSG) mice to establish pulmonary fibrosis models and found a significant regulatory effect of PD-L1 on invasive fibroblasts [21]. Additionally, an elevation in PD-L1 levels was observed in a humanized mouse model [76].

In addition to the abovementioned mouse models of bleomycin-induced lung fibrosis, transgenic techniques, and silica can also be utilized to establish mouse models of pulmonary fibrosis for the evaluation of PD-1/PD-L1 expression. Cui et al. detected elevated levels of PD-L1 expression in the lung tissue of IL-6 knockout mice, which is consistent with the results observed for PD-L1 in bleomycin-modelled lung fibrosis mice [76]. Zhao et al. found abnormal levels of expression of immune checkpoint molecules on these cells in a mouse model of silica-induced lung fibrosis, ultimately leading to systemic immune dysregulation [83]. Li et al. observed the same phenomenon as in BLM-induced pulmonary fibrosis in a mouse model of paraquat-induced pulmonary fibrosis, with upregulation of PD-L1 expression in lung tissue [74]. In summary, the aberrant expression levels of PD-1 and PD-L1 in certain lung cell types in a mouse model of pulmonary fibrosis suggest that PD-1/PD-L1 may play a role in the process of pulmonary fibrosis.

We observed differences in the animal models used in these studies. BLM-induced mouse pulmonary fibrosis model is the most commonly used animal model for the study of IPF, which can demonstrate the pathogenesis of human IPF [84], but it has certain limitations because its fibrosis resolves spontaneously after 28 days [85] The use of Silica in the lungs also causes persistent fibrosis, but is more similar to silicosis in humans than pulmonary fibrosis [85]. Systemic administration of paraquat causes fibrosis-like changes in the lungs of animals, but other organs (e.g., liver, kidney, etc.) also fail, increasing mortality rates [86]. Humanized mouse models can better elucidate the mechanism of human fibroblasts inducing pulmonary fibrosis in vivo, but the main issue is the availability of cells from human patients with IPF [87]. Although different modeling methods were used, the results showed increased expression levels of PD-1/PD-L1, suggesting that PD-1/PD-L1 was involved in the process of pulmonary fibrosis. Therefore, the researchers conducted further studies.

Current studies have shown that the fibrotic process is a combination of multiple cells, growth factors, and fibrosis-associated proteins, and their interactions determine the outcome of fibrosis [88]. The role of PD-1/PD-L1 in IPF can be explored from two perspectives.

**Fig. 2:**
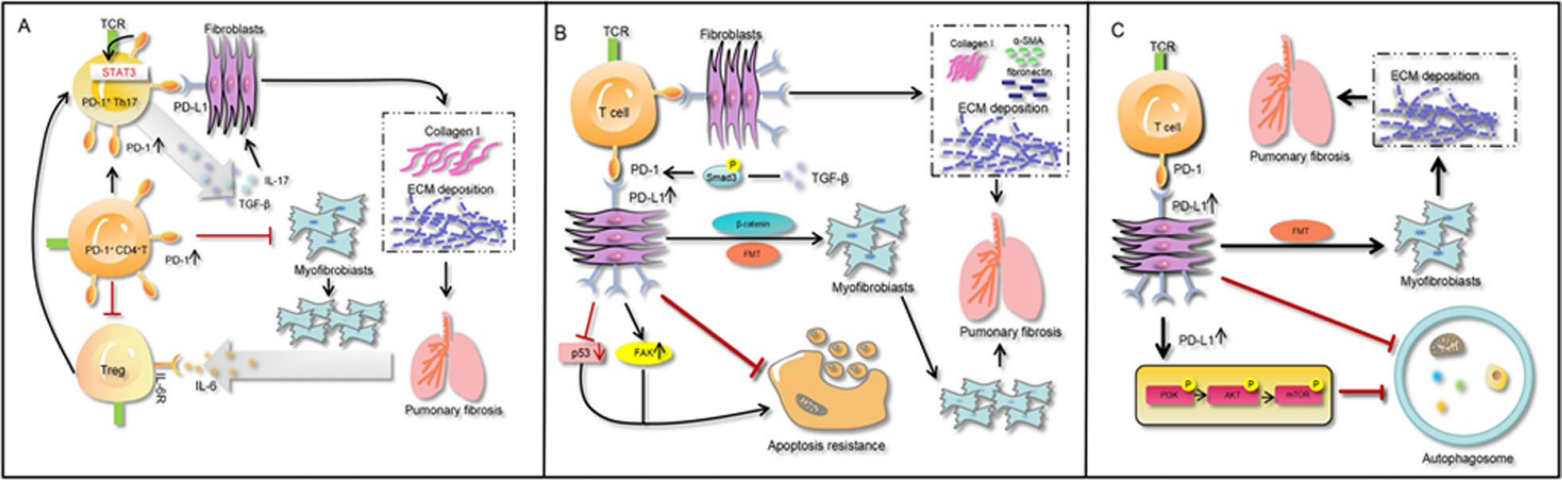
Cellular and molecular mechanisms of PD-1/PD-L1 involvement in the pathogenesis of IPF. **A** PD-1 mediates the up-regulation of IL-17 and TGF-β production by PD-1^+^Th17 cells through STAT3, which promotes lung fibrosis, and PD-1 inhibits the differentiation of CD4^+^T cells to Treg cells, which promotes the production of type I collagen and inhibits myofibroblast proliferation; **B** PD-L1 on lung fibroblasts inhibits myofibroblast proliferation by inhibiting the p53 pathway and activating the FAK pathway, causing myofibroblasts to evade phagocytosis, leading to excessive proliferation of myofibroblasts, resulting in lung fibrosis. In addition, PD-L1 mediates lung fibroblast-to-myofibroblast transformation (FMT) through Smad3 and β-catenin signaling pathways, thus promoting lung fibrosis; **C** PD-L1 upregulation on lung fibroblasts promotes fibrosis by inhibiting autophagy leading to myofibroblast proliferation and ECM deposition

Mechanistic experiments first recognized that PD-1^+^CD4^+^ T cells can promote pulmonary fibrosis and TGF-β production mainly through IL-17A, where T helper 17 (Th17) cells are the main CD4^+^ T cell subset expressing TGF-β. In an in vivo animal experiment, blocking PD-1 expression significantly reduced the expression of STAT3, IL-17A, and TGF-β on Th17 cells, thereby reducing the production of type I collagen by fibroblasts and attenuating lung fibrosis [67]. Similar results were observed in another study [78]. In addition, a study confirmed that maintaining the functional homeostatic balance between Th17/Treg cells is an important part of regulating autoimmunity and treating cancer [89]. Tregs can up-regulate the expression of TGF-β [90], and IL-6 allows Treg to regain the characteristics of Th17 cells [91]. Elevated IL-6 levels were also observed in patients with IPF. Therefore, we hypothesized that elevated IL-6 leads to the subpopulation conversion of Treg to Th17 cells, which increases the production of IL-17 and TGF-β and promotes lung fibrosis (Fig. 2A). Another study indicated that an increase in PD-1/PD-L1 inhibited CD4^+^ T cell differentiation to Treg cells, thereby reversing the effect of TGF-β on Treg cell differentiation. PD-1 promoted type I collagen production in myofibroblasts while decreasing the proliferation of myofibroblasts co-cultured with CD4^+^ T cells [69] (Fig. 2A). Upregulation of PD-L1 expression on fibroblasts has also been observed in studies on lung fibrosis, especially in invasive fibroblasts, which can promote lung fibrosis [21, 23, 76, 77]. We next explored the mechanism of the involvement of PD-L1 in IPF in fibroblasts.

Research suggests that high expression of PD-L1 on lung fibroblasts can be regulated through the p53 and FAK pathways. Knockdown of PD-L1 in invasive fibroblasts attenuates the severity of lung fibrosis in lung fibroblasts and mice [21]. Evidence also supports that p53 expression levels in myofibroblasts are significantly lower than those in normal lung fibroblasts [92, 93]. PD-L1 knockdown in IPF fibroblasts and targeting of PD-L1 by FAK inhibition or PD-L1-neutralizing antibodies blunts invasion and attenuates fibrosis [21]. In addition, the acquisition of p53 function could sensitize lung fibroblasts to apoptosis and prompt macrophages to clear apoptotic myofibroblasts, thereby attenuating pulmonary fibrosis symptoms in a mouse model of pulmonary fibrosis [93]. Therefore, PD-L1 on lung myofibroblasts may lead to IPF by inhibiting the p53 pathway, leading to myofibroblast resistance to apoptosis, and inhibiting phagocytosis by macrophages, leading to overproliferation of myofibroblasts (Fig. 2B). Recently research suggests that knockdown of PD-L1 in primary human lung fibroblasts significantly attenuated the expression of myofibroblast markers induced by TGF-β. TGF-β could induce PD-L1 to interact with Smad3, suggesting that PD-L1 may act as a cofactor for Smad3 to promote the process of lung fibrosis. PD-L1 knockdown attenuated TGF-β-induced GSK3β phosphorylation/inhibition and β-catenin upregulation, suggesting PD-L1-mediated GSK3β/β-catenin signaling in FMT [23]. Another study also showed a relation between the GSK3β/β-catenin signaling pathway and the progression of IPF [90]. Thereby, PD-L1 in lung fibroblasts was upregulated to promote the development of IPF through the Smad3 pathway and the β-catenin pathway (Fig. 2B).

Autophagy is also involved in the development of IPF. Inhibition of autophagy can induce myofibroblast proliferation and ECM deposition, leading to pulmonary fibrosis [94, 95]. The application of autophagy activators can attenuate the extent of pulmonary fibrosis [96]. It was found that anti-PD-L1 monoclonal antibody down-regulated the PI3K/AKT/mTOR pathway to induce autophagy, thereby inhibiting TGF-β1-induced lung fibroblast invasiveness and ECM deposition [77]. Inhibition of PI3K inhibits AKT phosphorylation and TGF-β-induced proliferation of fibroblasts as well as expression of markers in myofibroblasts [97]. Further, mTOR acts as a downstream molecule of the PI3K/AKT pathway, and inhibition of the PI3K/AKT pathway inhibits this target, upregulates myofibroblast autophagy and attenuates lung fibrosis [98] (Fig. 2C).

The mechanism of action of the PD-1/PD-L1 axis in IPF is not yet fully elucidated and further exploration is needed. We summarize the results of current PD-1/PD-L1 inhibitors in IPF studies below to explore the feasibility of targeting PD-1/PD-L1 for the treatment of IPF.

The majority of studies revealed a reduction in the extent of lung tissue lesions with anti-PD-L1 mAb in mouse models of pulmonary fibrosis [21, 67, 76, 77, 83]. Cui et al. observed that pulmonary fibrosis was reduced in a mouse fibrosis model after blocking PD-L1, thus providing a potential treatment strategy for patients with pulmonary fibrosis [76]. By establishing a humanized severe combined immunodeficiency IPF model, Geng et al. suggested that targeting PD-L1 during the early and late stages might significantly reduce the invasion of IPF fibroblasts and could not only eliminate the progression of pulmonary fibrosis but also reverse it [21]. Lu et al. found that anti-Pd-L1mAb ameliorated lung tissue disorganization and collagen deposition in mice with pulmonary fibrosis while demonstrating that it possesses the ability to activate autophagy in pulmonary fibrosis [77]. Zhao et al. conducted a similar study on silica-induced pulmonary fibrosis in mice and discovered that PD-1/PD-L1 signal inhibition significantly improved silicosis [83]. Current research is not limited to single-drug studies of PD-1/PD-L1 pathway inhibitors, and some researchers are interested in combination therapy. A study combined pirfenidone with a PD-L1 blocker to explore its treatment effects in mouse models of pulmonary fibrosis and LC [99]. Combined therapy with pirfenidone and PD-L1 blockers promoted immune cell invasion and inhibited tumor outgrowth while improving the prognosis of mice. This combination treatment regimen reduced lung fibrosis in mice. Some studies have also pointed out that human MSCs contribute to immune regulation, which has been used in several preclinical studies on IPF treatment [100]. The efficacy of ICIs in the aforementioned mouse models of pulmonary fibrosis demonstrates that PD-L1 inhibitors have a promising effect on alleviating pulmonary fibrosis.

The researchers also designed in vitro experiments to determine whether inhibition of PD-L1 reduces lung fibrosis. The in vitro experiments demonstrated that blocking the PD-1/PD-L1 pathway can inhibit STAT3 expression in CD4 + T cells, leading to low expression of IL-17A and TGF-β [67]. A previous report showed that CD274 knockdown in IPF fibroblasts by FAK inhibition or CD274-neutralizing antibodies, reduced invasion and alleviated fibrosis, whereas PD-L1 expression on invasive fibroblasts promoted lung fibrosis [21]. Inhibition of PD-1 appears to be less effective than inhibition of PD-L1 in preclinical IPF models, which may be related to the genetic ablation of PD-1 [67]. Given the elevated levels of immune checkpoint expression in patients with IPF, novel ICIs may provide new immunotherapeutic options.

The above preclinical studies have shown that inhibition of immune checkpoint pairs can attenuate the progression of pulmonary fibrosis, and that inhibition of PD-L1, in particular, can produce an antifibrotic effect. However, PD-1/PD-L1 inhibitors cannot be assumed to directly lead to results for the clinical treatment of IPF. While the fibrotic process is a complex process in which antifibrotic mediators interact with pro-fibrotic mediators in a microenvironment composed of different cell types [76], immune tolerance imbalances and the occurrence of immune-related adverse events (irAEs) require attention. Therefore, although preclinical studies have shown that targeting PD-1/PD-L1 immune checkpoints attenuates pulmonary fibrosis, more evidence is needed to support how these preclinical studies can be transferred to practical clinical applications.

### Immune-related adverse events: a barrier to IPF immunotherapy?

ICIs, while enhancing the normal immune response, may enhance the anti-tumor effects of cellular immunity, leading to an immune tolerance imbalance and immune-related adverse events (irAEs). IrAEs can involve all organs throughout the body and are common, with data from one study showing that over 80% of patients using ICIs developed irAEs in some systems [101, 102]. In the targeted treatment of ICIs for LC, immunosuppressant-related pneumonia requires consideration. Studies have found that the use of ICIs in patients with LC with ILD is associated with a higher risk of developing immune checkpoint inhibitors related pneumonitis (CIP) than that in patients without ILD, but these studies suggest that CIP is not associated with increased mortality [103–105]. The response rate, progression-free survival, and overall survival of NSCLC patients with concomitant ILD treated with ICIs were similar to those of patients without ILD, suggesting that patients with NSCLS treated with ILD could also benefit from ICI treatment [103]. However, since the presence of interstitial pneumonia is currently regarded as the exclusion criterion for ICI clinical trials [106, 107], direct evidence is lacking to prove the effect of ICIs on pulmonary fibrosis symptoms. Currently, no studies have directly validated the utility of PD-1/PD-L1 inhibitors on pulmonary fibrosis, and conclusive experimental evidence to support their therapeutic value is scarce. Studies have shown that ICIs in patients with IPF combined with squamous cell carcinoma, the addition of nidanib may prevent drug-induced pneumonia or acute exacerbation of IPF [108]. Another study showed that pirfenidone combined with PD-L1 inhibitors in the treatment of tumor pulmonary fibrosis in mice significantly delayed tumor growth, reduced pulmonary fibrosis, and improved mouse survival. This suggests that pirfenidone could be used as an adjunct to immunotherapy in cancer treatment [99]. Therefore, the study of the potential mechanism of irAEs not only contributes to the immunotherapy of tumors but also plays an important role in the treatment of IPF. Whether the combination of ICIs and antifibrotic drugs (such as nidanib and pirfenidone) can delay the pathogenesis of IPF can be considered as a research direction. In addition to investigating whether ICIs can be used to improve disease progression in patients with IPF, future studies should also clarify its role in prognosis.

## Conclusions and outlook

With the gradual exploration of the pathogenesis of IPF, new progress has been made in the treatment of IPF. Nonetheless, room for progress before satisfactory efficacy can be achieved. Current experiments have confirmed that the PD-1/PD-L1 pathway can interact with various cell types and pathways and is involved in promoting fibrosis and immune regulation in IPF. Simultaneously, animal experiments have revealed that the application of PD-1/PD-L1 inhibitors reduces the symptoms of pulmonary fibrosis. In this summary review, we present the effects of PD-1/PD-L1 in IPF; ongoing research suggests that it may offer a novel direction for future IPF therapy. Nevertheless, the following problems remain and, hence, require further investigations:

First, most studies published to date have reported PD-1/PD-L1 expression levels in IPF and confirmed its effect on pulmonary fibrosis; furthermore, the application of PD-1/PD-L1 ICIs has been proven to reduce lung fibrosis. However, the results are concentrated on animals and preclinical studies are poorly supported by complete clinical evidence. We have summarized in this review the role of PD-1/PD-L1 in the pathogenesis of IPF, and we still have a long way to go to translate these mechanisms into effective anti-pulmonary fibrosis therapies. The development of prognostic animal models and ex vivo primary human lung tissue models can help us achieve the transition from experiments to clinical applications [109]. Because the presence of pulmonary fibrosis is currently an exclusion criterion for receiving immune checkpoint inhibitors for lung cancer in clinical trials, the selection of patients for clinical trials with ICIs is difficult. Therefore, a more in-depth analysis of the mechanism of immune checkpoint involvement in pulmonary fibrosis, an accurate search for key therapeutic targets, and accurate and verified predictors of IPF progression, to identify target patients for inclusion in the experiment, is a reliable choice. A full understanding of the molecular mechanism of pulmonary fibrosis and stratified evaluation of IPF patients are helpful to tailor treatment and evaluate prognosis, thus improving clinical efficacy. Referring to the research methods in chronic obstructive pulmonary disease, the reasonable stratification of IPF patients to explore the relationship between different factors and disease progression and prognosis is also conducive to the treatment of IPF [110]. In addition, the development of single-cell genomics methodologies [111], molecular imaging of fibrosis [112], and other technologies can help us better conduct precision medicine research and provide help for the future development of IPF immunotherapy.

An important contribution of certain cytokines [113], such as IL-17A [67], IL-6 [114], and IL-8 [115] in pulmonary fibrosis has been observed. Studies have shown that simultaneous blockade of IL-6, CD47, and PD-L1 attenuates pulmonary fibrosis and may be associated with enhanced phagocytosis of fibroblasts and elimination of inhibition of adaptive immunity [76].In patients with COVID-19 combined with pulmonary fibrosis, and IL-6 antibodies showed better efficacy against COVID-19-induced cytokine storm. Therefore, IL-6 antibodies may be of benefit to patients with IPF [116]. IL-8 promotes senescence of IPF fibrous mesenchymal progenitor cells (MPCs) and up-regulation of PD-L1, allowing IPF MPCs to evade clearing by immune cells and thus exerting a pro-fibrotic effect [115]. In addition to this, there have been some studies exploring the effect of the combination of CD47 and PD-L1 antibodies in IPF [117, 118]. Studying the interrelationship between cytokines and PD-1/PD-L1 in the pathogenesis of IPF, further elucidating the role of PD-1/PD-L1 in the pathogenesis of IPF, and then exploring how to apply PD-1/PD-L1 inhibitors in the clinical treatment of IPF are the directions we can proceed in the future.

Second, EMT is still regarded as an important link in the development of pulmonary fibrosis in IPF. BLM-induced upregulation of PD-L1 was found to lead to EMT progression and fibroblast-like morphological changes in AEC. Alveolar epithelial cells are the progenitor cells of fibroblasts in vivo [119], and inhibition of EMT in AEC suppresses pulmonary fibrosis [120]. PD-L1 can induce EMT in AECs to promote lung fibrosis by directly binding to vimentin and inhibiting vimentin ubiquitination. Vimentin promotes BLM- and PD-L1-induced fibrosis in Human Pulmonary Alveolar Epithelial Cells, thereby promoting lung fibrosis [74]. Vimentin, as an important stimulator of EMT, is also a key factor in lung fibrosis. Therefore, exploring the interrelationship between EMT-related proteins and PD-1/PD-L1 thereby further investigating the specific pathogenesis of IPF could also bring new insights for the treatment of IPF.

Third, as the role of macrophages in the process of lung fibrosis is revealed, the understanding of lung macrophages is becoming clearer [121, 122]. For instance, one study showed that PD-L1 produced an immunosuppressive cell phenotype by delivering negative signals to macrophages and that PD-L1 antibody application reversed the phenotype [123]. In recent years, research on PD-1/PD-L1 immune checkpoints and tumor-associated macrophages has opened up new perspectives for the treatment of tumors [121, 124–128]. Activation of DDR1 in macrophages was found to play a role in IPF through inflammatory vesicle activation and macrophage responses [129]. Jovanovic et al. found PD-L1 expression on alveolar macrophages in IPF and suggested that PD-L1-positive alveolar macrophages have a regulatory/inhibitory phenotype with reduced phagocytosis, thus promoting the development of pulmonary fibrosis [66]. Therefore, we can focus more on how PD-L1 regulates the role of macrophages, which in turn are involved in lung fibrosis, to obtain useful insights.

With the development of nanomedicine, researchers have begun to explore whether nanomedicine can successfully solve real clinical problems. With this in mind, researchers have devised various approaches that have looked to enhance the effectiveness of immunotherapy, such as the application of PD-1/PD-L1 drugs in tumors [130]. The current study found that preparing iron death inhibitors as inhalable nanomedicines for the treatment of IPF may result in better efficacy [131]. Nanodesigned carbon monoxide donors may also improve BLM-induced pulmonary fibrosis [132]. The feasibility of preparing PD-1/PD-L1 inhibitors as nanomedicines or adopting a nanomedicine approach to IPF also needs to be further explored. In addition, green nanomaterials deserve our attention because of their low cost, low pollution, and safety to the environment and human health [133, 134].

Additionally, an increasing number of studies have investigated the mechanisms behind the role of traditional Chinese medicine (TCM) in pulmonary fibrosis, and the material basis and therapeutic mechanism of TCM treatment for IPF have been preliminarily explored and revealed [135, 136]. Researches are beginning to focus on the relationship between TCM and PD-1/PD-L1 immune checkpoints [137, 138]. For instance, berberine is a Chinese medicinal ingredient that blocks PD-L1 expression in tumor cells and enhances the sensitivity of tumor cells to T cells. Berberine exerts its antitumor effects by degrading PD-L1 through a ubiquitin/proteasome-dependent pathway [137]. A previous study used oral Gancao Ganjiang decoction to treat bleomycin drip-induced pulmonary fibrosis in a mouse model and observed that this decoction reduced the PD-1 levels in lung tissues and the PD-1 expression in peripheral blood CD4^+^ T cells, thereby diminishing the inflammatory response and fibrosis in the lungs [78].

Most TCM components applied in IPF treatment have multiple targets, pathways, and levels of action. At the experimental stage, several active TCM ingredients have displayed tremendous value in IPF treatment, providing a reference for new treatment ideas for IPF. For example, Bufei huoxue capsule, as a TCM formulation, can alleviate BLM-induced pulmonary fibrosis in mice by inhibiting the TGF-β/Smad2/3 signaling pathway [139]. Considering the critical function of the PD-1/PD-L1 immune checkpoints in IPF, is it viable to treat IPF using the aforementioned nanomedicines and TCM? This question requires researchers to continue conducting further investigations. Using network pharmacology methods to design multi-target drug molecules for specific signal nodes, combining molecular docking technology with traditional experimental methods, can better help us understand the potential mechanism of IPF and the development of new drugs in the future.

In conclusion, ICIs, particularly PD-L1 inhibitors, may be effective as a treatment for IPF and warrant further exploration and development.

## Data Availability

No new data were created or analyzed in this study. Data sharing is not applicable to this article.
